# Dietary fatty acids promote gut health in weaned piglets by regulating gut microbiota and immune function

**DOI:** 10.3389/fmicb.2025.1558588

**Published:** 2025-04-09

**Authors:** Zongze Fan, Lei Lei, Xingyue Wu, Ronghui Xing, Pengfei Du, Ziyang Wang, Huijuan Zhao, Yanqun Huang, Wen Chen, Xuemeng Si

**Affiliations:** ^1^Institute of Animal Science and Technology, Henan Agricultural University, Zhengzhou, China; ^2^Zhengzhou Agricultural Comprehensive Administrative Law Enforcement Detachment, Zhengzhou, China; ^3^Delvigent (Hebei) Biotech Co. Ltd., Cangzhou, China

**Keywords:** medium-chain and short-chain fatty acids, zinc oxide, oxidative stress, inflammatory, *Lactobacillus*, weaned piglets

## Abstract

**Background:**

Post-weaning diarrhea in piglets is a common challenge that adversely impacts growth performance and increases mortality, leading to severe economic losses. Medium-chain fatty acids (MCFA) and short-chain fatty acids (SCFA) are frequently used as feed additives due to their bioactive properties. This study evaluated the effects of two different blends of MCFA and SCFA (VSM and VS + VM) as alternatives to zinc oxide (ZnO) on growth performance, nutrient digestibility, oxidative stress, inflammatory, and gut microbiota composition in weaned piglets.

**Methods and results:**

A total of 108 piglets (8.22 ± 0.51 kg) were randomly assigned to three treatments: control (CON, basal diet + ZnO), VSM (basal diet + higher MCFA and lower SCFA content) and VS + VM (basal diet + higher SCFA and lower MCFA content). Results indicated that Both VSM and VS + VM, can replace ZnO to relieve diarrhea of weaned piglets as evidenced by increased average daily gain (ADG) and decreased feed to gain ratio (F/G) in 1–15 days, with no difference in final body weight compared to the CON group. Additionally, dietary MCFA and SCFA supplementation improves anti-oxidative and anti-inflammatory capacity by decreased of malondialdehyde (MDA) activity, and inhibited proinflammatory cytokine tumor necrosis factor α (TNF-α) and interleukin (IL-1β, IL-17A) secretion. Further study showed that the protective effect of MCFA and SCFA were associated with restoring gut barrier, upregulating abundances of *Lactobacillus* and *Roseburia* of piglets.

**Interpretation:**

Collectively, the combination of MCFA and SCFA alleviated oxidative stress, modulated inflammation, and supported gut barrier function in weaned piglets, offering a promising alternative to ZnO, with VSM showing superior effects.

## Introduction

Weaning is widely recognized as the most critical and stressful stage in piglet development. Following weaning, piglets are exposed to a combination of environmental and psychological stressors, often resulting in reduced growth performance, disruption of the gut microbiota, and the onset of diarrhea (Laine et al., 2008). A primary factor contributing to post-weaning diarrhea is oxidative stress, which arises from the imbalance between pro-oxidative forces and antioxidant defenses (Qiao et al., 2023). The imbalance compromises the gut barrier, leading to increased permeability and facilitating pathogen translocation (Feng et al., 2020). The resulting local and systemic inflammation exacerbates diarrhea and further hinders intestinal function, intensifying the negative impacts on piglet health and growth (Campbell et al., 2013). To mitigate the negative effects of post-weaning stress, high doses of zinc oxide (ZnO) have traditionally been included in piglet diets due to their multiple benefits (Long et al., 2017). ZnO supplementation enhances intestinal development, increases digestive enzyme activity and strengthens the intestinal barrier functionality (Liu et al., 2014). Additionally, ZnO demonstrates antimicrobial properties that contribute to modulating gut microbiota composition (Tang et al., 2024). Although ZnO supplementation are effective in reducing diarrhea and supporting growth, their long-term use raises concerns, whereas high levels of ZnO in animal feed have contributed to environmental contamination (Grilli et al., 2015; Jensen et al., 2016; Tan et al., 2023). Thus, the European Union implemented a ban on the inclusion of medicinal doses of ZnO in animal feeds as of June 2022 (Bonetti et al., 2021), thereby reducing environmental concerns and the increasing regulatory restrictions on ZnO usage, alternatives such as organic acids, probiotics, and plant-derived bioactive compounds have gained attention (Abd El-Hack et al., 2022).

Medium-chain and short-chain fatty acids (MCFA/SCFA) have gained popularity as feed additives due to their critical roles in promoting intestinal health and enhancing the growth performance of piglets (Hu et al., 2023). MCFA, consist of fatty acids with 6–12 carbon atoms, are mainly composed of caprylic acid (C8:0), capric acid (C10:0), and lauric acid (C12:0). Compared to conventional organic acids, MCFA possess potent antibacterial activity, particularly against Gram-positive bacteria and *Escherichia coli*, thereby contributing to the regulation of balance of intestinal microbiota (Jiao et al., 2023). Additionally, MCFA can be rapidly absorbed by the organism through the portal vein, and utilized by the liver without requiring the carnitine transport system to enter mitochondria (Nagao and Yanagita, 2010). This enables them to directly supply energy to the intestinal epithelium, thereby promoting the renewal and repair of enterocytes, which is crucial for maintaining intestinal integrity (Zentek et al., 2011). SCFA, on the other hand, are important metabolites produced by intestinal microbiota and serve as the primary energy for colon epithelial cells (den Besten et al., 2013; Nakkarach et al., 2021). SCFA are organic acids containing 1–6 carbon atoms, such as formic acid, acetic acid, and propionic acid, which play a crucial role in maintaining intestinal health (Nakkarach et al., 2021). MCFA and SCFA play an important role in regulating gastrointestinal pH, improving the activity of digestive enzymes, and promoting the growth of acid-tolerant microorganisms, such as lactic acid-producing bacteria (Lauridsen, 2020).

Fatty acids with distinct chain lengths exhibit differential biological properties and mechanisms of action, thereby generating synergistic effects when supplemented in combination (López-Colom et al., 2020). For example, MCFA can disrupt the microbial cell wall, allowing SCFA to enter the bacterial cytoplasm and exert antibacterial effects (Szabó et al., 2023). A study revealed that combined supplementation of capric acid and SCFA in piglets diets resulted in enhanced growth performance compared to SCFA alone (Hanczakowska et al., 2013). Additionally, another study demonstrated that a blend containing MCFA and butyrate supplementation improved antioxidant capacity, optimized intestinal morphology, and reduced diarrhea incidence in weaning piglets (Cai et al., 2024). Despite these promising findings, research on the optimal ratio of MCFA to SCFA remains limited, and further studies are needed to determine the most effective formulation for improving piglet health and performance. In earlier studies, mixtures with higher SCFA than MCFA added to piglet diets were shown to improve growth performance (Kuang et al., 2015). However, our product (VSM) contains a higher MCFA and lower SCFA content, which has been validated in practical production settings to enhance piglet growth performance. To explore the most effective combination, we formulated a VS + VM group, where SCFA levels were higher than MCFA. This allowed us to compare the two fatty acid ratios and determine which formulation yields better growth and gut health outcomes.

The study was conducted to investigate the effects of ZnO substitute MCFA and SCFA supplementation on growth performance, redox homeostasis, and gut microbiota composition of weaned piglets. Specifically, we investigate the protective roles of MCFA and SCFA in mitigating weaning-induced stress and explore potential mechanisms underlying their effects.

## Materials and methods

### Animal care

All animal procedures were approved by the Institutional Animal Care and Use Committee of Henan Agricultural University (Approval No. HNND20190612) and were conducted in accordance with the Guide for the Care and Use of Laboratory Animals.

### Reagents

MCFA and SCFA products were supplied by Delvigent (Hebei) Biotech Co. Ltd., Cangzhou, China. Details of the product composition are as follows:

Phase 1: VSM group: MCFA (caprylic acid, capric acid, and lauric acids) at 1.25 kg/t, SCFA (0.5 kg/t formic acid, 0.19 kg/t acetic acid, and 0.15 kg/t propionic acid). The MCFA:SCFA ratio was approximately 1.5:1. VS + VM group: MCFA at 1.16 kg/t, SCFA (1.24 kg/t formic acid 0.19 kg/t acetic acid), 0.1 kg/t lactic acid. The MCFA:SCFA ratio was approximately 0.8:1.
Phase 2: VSM group: MCFA (caprylic acid, capric acid, and lauric acids) at 1.00 kg/t, SCFA (0.4 kg/t formic acid, 0.15 kg/t acetic acid, and 0.12 kg/t propionic acid), the MCFA:SCFA ratio was approximately 1.5:1. VS + VM group: MCFA at 0.87 kg/t, SCFA (1.24 kg/t formic acid 0.19 kg/t acetic acid) 0.1 kg/t lactic acid, the MCFA:SCFA ratio was approximately 0.6:1.

The fatty acid products have silica as their carrier, the common organic acids in this study comprise citric acid, benzoic acid, fumaric acid, and lactic acid.

### Animals and experiment design

A total of 108 Duroc × Landrace × Large Yorkshire weaned piglets (28 days old, 8.22 ± 0.51 kg) were randomly assigned to one of three diet treatments in a 2-phase feeding trial (nine piglets per replicate, balanced for sex). Dietary treatments were as follows:

In phase 1 (1–15 days), CON (basal diet + 2 kg/t ZnO + 8 kg/t common organic acid), VSM (basal diet + higher MCFA and lower SCFA content, the MCFA/SCFA ratios were 1.5:1) and VS + VM (basal diet + higher SCFA and lower MCFA content, the MCFA/SCFA ratios were 0.8:1). In phase 2 (16–35 days), CON (basal diet + 7 kg/t common organic acid), VSM (basal diet + higher MCFA and lower SCFA content, the MCFA/SCFA ratios were 1.5:1) and VS + VM (basal diet + higher SCFA and lower MCFA content, the MCFA/SCFA ratios were 0.6:1).

The detailed composition and nutritional levels of the experimental diet are presented in Table 1. Each pen was equipped with a slatted floor, and environmental conditions were strictly regulated. The temperature inside the pig housing facility was maintained at 22°C–26°C, with a humidity level of 60–70%. Piglets were provided ad libitum access to feed and water throughout the study. Fecal samples were collected on day 35, frozen in liquid nitrogen, and stored at −80°C. Blood samples were collected from the anterior vena cava, and serum was separated and stored at −20°C for subsequent analysis.

**Table 1:** Ingredient composition and analyzed nutrient concentration of experimental diets (%, as—dry matter basis).

Items	Contents	
	1–15 days	16–35 days
Diet composition		
Broken rice	19	0
Corn	28	52
Puffed corn	12	0
Expanded soybeans	4	10
Fish meal	5	5
Peeled soybean meal	6	12
Fermented soybean meal	8	4
Whey powder	3	6
Lactose	3	0
Sucrose	4	2
Glucose	2	2
Soybean oil	1	2
4% piglet premix	4	4
Nutrient composition		
Digestible energy, Mcal/kg	3.53	3.44
Metabolic energy	3.14	3.08
Crude protein, %	18.01	17.63
Crude fat	4.20	4.07
Crude fiber	2.00	2.03
Coarse ash content	4.20	4.75
Calcium	0.54	0.65
Total phosphorus	0.52	0.55
Non phytic acid phosphorus	0.36	0.39
Lysine	1.60	1.58
Threonine	1.05	1.02
Methionine	0.56	0.57
Cysteine	0.80	0.80
Valine	1.00	0.96
Arginine	1.20	1.10
Isoleucine	0.90	0.85

Provided the vitamin and minerals as following per kilogram of diet: 1,800 IU vitamin A as retinyl acetate; 200 IU vitamin D3 as cholecalciferol; 11 IU vitamin E as DL-a-tocopherol acetate; 0.5 mg vitamin K3 as menadione; 1 mg vitamin B1; 3.5 mg vitamin B2; 10 mg vitamin B5; 1.5 mg of vitamin B6; 17.5 mg vitamin B12; 15 mg niacin; 0.3 mg folic acid; 0.5 mg choline 0.05 mg biotin; 105 mg Fe; 6 mg Cu; 110 mg Zn; 4 mg Mn; 0.30 mg Se; 0.14 mg I.

### Growth performance

Piglets were weighed at the beginning of the experiment and at the end of each phase. Feed intake were recorded daily. Diarrhea was assessed daily using the following scoring system: 0: normal, well-formed feces (solid or in soft strips) without excess moisture or mucus; 1: mild diarrhea, soft, partially formed feces; 2: moderate diarrhea, semi-liquid and mobile feces; 3: severe diarrhea, totally watery feces without any solid content.


$$\begin{array}{l} \mathrm{Diarrhea rate}\;\left(\%\right)=\left(\mathrm{Total number of diarrhea in each group}\right)\\ /\left(\mathrm{Total number of piglets in each group}\times \mathrm{Trial days}\right)\times 100\% \end{array}$$

Diarrhea index=Total fecal score/Total piglets in each group

### Nutrient digestibility

Apparent total tract digestibility (ATTD) of nutrients was determined using ash insoluble in hydrochloric acid as an endogenous marker. Fecal samples were thawed, dried at 65°C for 72 h, diets and fecal samples were accurately weighed for AIA analysis [Method 942.05; (AOAC, 2007)]. and analyzed for CP [Method 988.05; (AOAC, 2007)], EE [Method 920.39; (AOAC, 2007)], and ash content. Diets and ingredients were also analyzed for Ca [Method 942.05; (AOAC, 2007)] and P [Method 965.17; (AOAC, 2007)]. ATTD values for EE, CP, Ca, and P were calculated as follows:


$$\mathrm{ATTD}\;\left(\%\right)=1-\left(\left[N_{f}\times A_{d}\right]/\left[N_{d}\times A_{f}\right]\right)$$

where *N*_f_ is nutrient concentration in feces, *N*_d_ is nutrient concentration in diet, *A*_d_ is AIA concentration in diet, and *A*_f_ is AIA concentration in feces.

### Serum inflammatory cytokines and antioxidant

Serum biochemical indexes, including total protein (TP), albumin (ALB), globulin (GLB), glucose (GLU), triglyceride (TG), total cholesterol (TC), alkaline phosphatase (ALP) and blood urea nitrogen (BUN), were determined using a BK280 Automatic Biochemical Analyzer (Shandong, China). Intestinal permeability indexes, including D-lactate and diamine oxidase (DAO) were determined by their specific assay kits (MM-33732O1, MM-0438O2) Jiangsu Meimian Industrial Co., Ltd., China; serum antioxidant parameters, including total superoxide dismutase (T-SOD), total antioxidant capacity (T-AOC), glutathione peroxidase (GPX), catalase (CAT), malondialdehyde (MDA), hydrogen peroxide (H_2_O_2_), were determined by their specific assay kits (A001-3-1, A015-2-1, A005-1-2, A007-1-1, A003-1-2, A064-1-1) from Nanjing Jiancheng Bioengineering Institute (Nanjing, China) inflammatory cytokines, including interferon-γ (IFN-γ), transforming growth factor-β (TGF-β), tumor necrosis factor-α (TNF-α), interleukin (IL-1β, IL-4, IL-6, IL-10, and IL-17A) were determined using ELISA kit (MM-0412O2, MM-36525O2, MM-0383O2, MM-0422O2, MM-0419O2, MM-0418O2, MM-0425O2, MM-77840O2) from Jiangsu Meimian Industrial Co. Ltd., China.

### Gut microbiota analysis

Microbial genomic DNA was extracted from piglet feces using DNA stool mini kit (Tiangen, Beijing, China) following the manufacturer’s instructions. DNA concentration was quantified using a Nanodrop spectrophotometer, and quality was assessed by 1.2% agarose gel electrophoresis. The V3–V4 regions in 16S rDNA genes were amplified by specific primers (338 F and 806 R). The obtained PCR products were purified using the AxyPrep DNA Gel Extraction Kit (Axygen Biosciences, Union City, CA, United States), and sequencing was performed on an Illumina MiSeq platform (Personal Biotechnology Co., Ltd., Shanghai, China).

We performed standardized bioinformatics analyses on the raw data (FASTQ format) generated from Illumina paired-end sequencing to ensure the accuracy and reproducibility of the results. First, the raw FASTQ files were imported into the QIIME 2 (2019.4) platform and converted into a format compatible with QIIME 2 (qza format). Subsequently, the qiime cutadapt trim-paired plugin was used to remove primer sequences from the reads, ensuring the precise removal of primer fragments and filtering out reads that did not match the primer sequences, thereby improving data quality. Next, the qiime dada2 denoise-paired plugin was employed to call DADA2 for denoising. This step applied stringent quality control parameters to filter out low-quality sequences. Chimera sequences were identified and removed using the UCHIME algorithm (v8.1), effectively eliminating potential artifacts that could impact downstream analyses. This process generated high-confidence amplicon sequence variants (ASVs) and their associated abundance tables after chimera removal.

To analyze the microbiota composition, stacked bar plots were generated using the ggplot2 package (version 2.2.1) in R. Correlation network was constructed using the igraph package (version 1.1.2) to visualize relationships between bacterial taxa and host immune markers. Inter-group Venn analysis was conducted with the VennDiagram package (version 1.6.16) to identify overlapping and unique taxa among treatment groups. Given the compositional and non-parametric nature of microbiome data, statistical comparisons of microbial abundance were conducted using Kruskal–Wallis tests followed by *post hoc* Dunn’s multiple comparisons to identify significant differences between groups. Additionally, Linear Discriminant Analysis Effect Size (LEfSe) was applied to detect bacterial taxa that were significantly enriched in each treatment group. The alpha-diversity index (Chao1, Observed species, Faith PD, and Goods coverage) and beta-diversity (PCoA analysis based on Bray_curtis) were computed using QIIME2. Alpha diversity analysis was assessed using the Kruskal-Walli’s test. Spearman correlation coefficients of microbial taxa were calculated and visualized using the heatmap package (version 2.3.1) in R software.

### Statistical analysis

Data were statistically analyzed by GraphPad Prism 8.0. one-way ANOVA was used to assess differences among treatment groups when data followed a normal distribution. In cases where normality was not met, Kruskal–Wallis tests were applied to ensure robust statistical comparisons. Differences between treatments were determined by Duncan’s post hoc tests and *p* ≤ 0.05 was taken to indicate statistical significance.

## Results

### Growth performance

The effects of dietary supplementation of medium and short-chain fatty acids on growth performance of piglets are summarized in Table 2. Body weight at 15 days and 35 days did not differ among the three groups of piglets. From days 1 to 15, piglets fed the VS + VM diet showed a significantly higher ADG and a lower F/G compared with the CON group (*p* < 0.05), and VSM group showed a trend higher ADG. However, during the second phase (days 16 to 35), the ADG of piglets in the VS + VM group significantly reduced compared to CON (*p* < 0.05).

**Table 2:** Effects of MCFA and SCFA on growth performance and diarrhea rate of weaned piglets.

Items	Treatments			*p*-value
	CON	VSM	VS + VM	
Initial BW, kg	8.33 ± 0.52	8.21 ± 0.68	8.12 ± 0.41	0.866
15 days BW, kg	11.83 ± 0.51	12.06 ± 0.92	12.50 ± 0.60	0.429
Final BW, kg	21.96 ± 1.36	21.22 ± 1.50	20.29 ± 0.22	0.114
1 day to 15 days				
ADG (grams/day)	228.50 ± 15.84^b^	255.75 ± 20.25^ab^	288.00 ± 35.92^a^	0.028
ADFI (grams/day)	455.50 ± 48.34	442.25 ± 27.22	435.25 ± 4.57	0.515
F/G	2.03 ± 0.29^a^	1.75 ± 0.10^ab^	1.55 ± 0.19^b^	0.010
Diarrhea rate %	3.70 ± 1.65	2.75 ± 2.08	2.52 ± 1.15	0.592
Diarrhea index	0.84 ± 0.37	0.68 ± 0.55	0.52 ± 0.24	0.587
16 days to 35 days				
ADG (grams/day)	507.00 ± 46.81^a^	455.00 ± 42.43^ab^	389.75 ± 32.76^b^	0.010
ADFI (grams/day)	941.25 ± 78.97	885.50 ± 58.79	834.25 ± 20.02	0.100
F/G	1.85 ± 0.13	1.98 ± 0.10	2.15 ± 0.21	0.059
Diarrhea rate %	3.25 ± 1.46	3.37 ± 0.92	2.56 ± 1.01	0.580
Diarrhea index	1.09 ± 0.49	1.17 ± 0.36	0.83 ± 0.30	0.468
1 day to 35 days				
ADG (grams/day)	386.00 ± 33.32	367.00 ± 29.72	345.50 ± 11.82	0.155
ADFI (grams/day)	731.00 ± 67.01	690.25 ± 43.41	661.50 ± 10.47	0.122
F/G	1.88 ± 0.15	1.90 ± 0.08	1.90 ± 0.12	0.943
Diarrhea rate %	3.44 ± 1.49	3.15 ± 1.14	2.54 ± 0.58	0.538
Diarrhea index	1.92 ± 0.85	1.87 ± 0.72	1.35 ± 0.30	0.439

BW, body weight; ADFI, average daily feed intake; ADG, average daily gain; F/G, feed to gain ratio; CON, basal diet + ZnO/common organic acid; VSM, basal diet + higher MCFA and lower SCFA content; VS + VM, basal diet + higher SCFA and lower MCFA content. Different letters mean statistically significant difference among the groups (*p* < 0.05). The data are presented as the means ± SD.

### Nutrient digestibility

As shown in Table 3, the dietary supplementation of medium- and short-chain fatty acids significantly affected nutrient digestibility. Compared with CON group, VSM treatment significantly increased the apparent digestibility of EE in 35 days, and VS + VM treatment significantly improved the apparent digestibility of Ca in 35 days (*p* < 0.05).

**Table 3:** Effects of MCFA and SCFA on nutrient digestibility of weaned piglet.

Items	Treatments			*p-*value
	CON	VSM	VS + VM	
15 days				
CP %	73.75 ± 9.36	81.00 ± 3.30	75.47 ± 4.57	0.286
EE %	54.26 ± 15.14	67.56 ± 6.98	55.85 ± 8.66	0.221
Ash %	41.83 ± 12.25	57.49 ± 8.50	49.30 ± 5.09	0.102
Ca %	49.33 ± 21.66	70.06 ± 13.70	67.32 ± 7.37	0.174
P %	40.43 ± 14.76	57.83 ± 10.10	53.35 ± 10.45	0.158
35 days				
CP %	75.78 ± 3.49	79.42 ± 2.24	77.20 ± 3.76	0.323
EE %	61.00 ± 4.60^b^	71.18 ± 3.39^a^	67.40 ± 4.77^ab^	0.025
Ash %	41.54 ± 5.85	43.65 ± 1.62	52.96 ± 10.01	0.087
Ca %	35.32 ± 16.55^b^	31.50 ± 3.05^b^	63.96 ± 11.43^a^	0.014
P %	44.63 ± 7.31	51.75 ± 3.14	48.97 ± 12.17	0.508

CP, crude protein; EE, crude fat; ash, crude ash; Ca, calcium; P, phosphorus; CON, basal diet + ZnO/common organic acid; VSM, basal diet + higher MCFA and lower SCFA content; VS + VM, basal diet + higher SCFA and lower MCFA content. Different letters mean statistically significant difference among the groups (*p* < 0.05). The data are presented as the means ± SD (*n* = 4).

### Serum biochemical parameters

**Figure 1:**
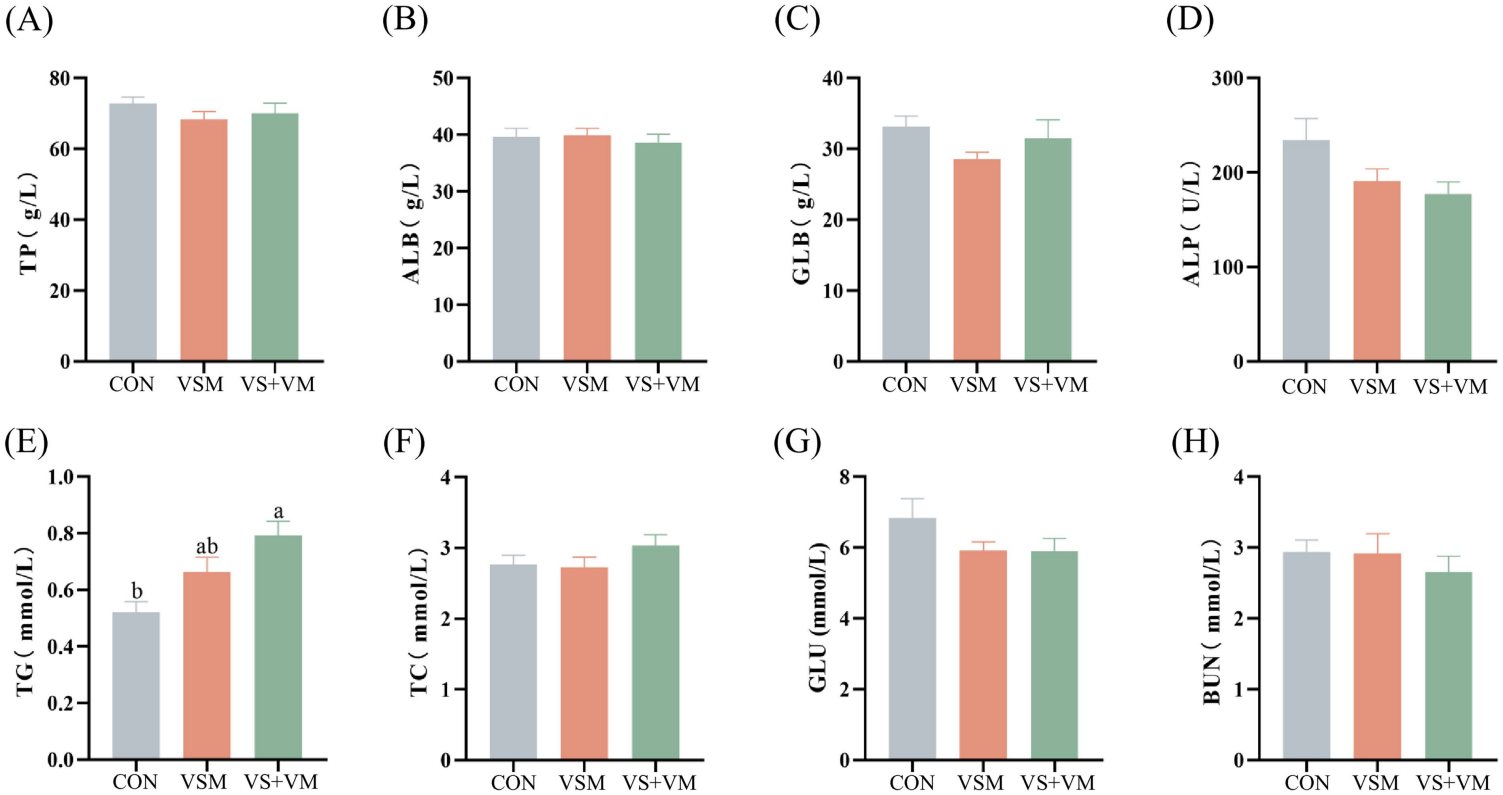
The effect of MCFA and SCFA addition on serum biochemical indexes of weaned piglets. **(A)** TP, **(B)** ALB, **(C)** GLB, **(D)** ALP, **(E)** TG, **(F)** TC, **(G)** GLU, and **(H)** BUN. Data were shown as means ± SEM (*n* = 8). TP, total protein; ALB, albumin; GLB, globulin; ALP, alkaline phosphatase; TC, total cholesterol; TG, total glyceride; GLU, glucose; BUN, blood urea nitrogen. CON, basal diet + ZnO/common organic acid; VSM, basal diet + higher MCFA and lower SCFA content; VS + VM, basal diet + higher SCFA and lower MCFA content. Different letters mean statistically significant difference among the groups (*p* < 0.05).

As shown in Figure 1, the VS + VM group had a significantly higher serum TG concentration than the CON group (*p* < 0.05).

### Intestinal permeability

**Figure 2:**
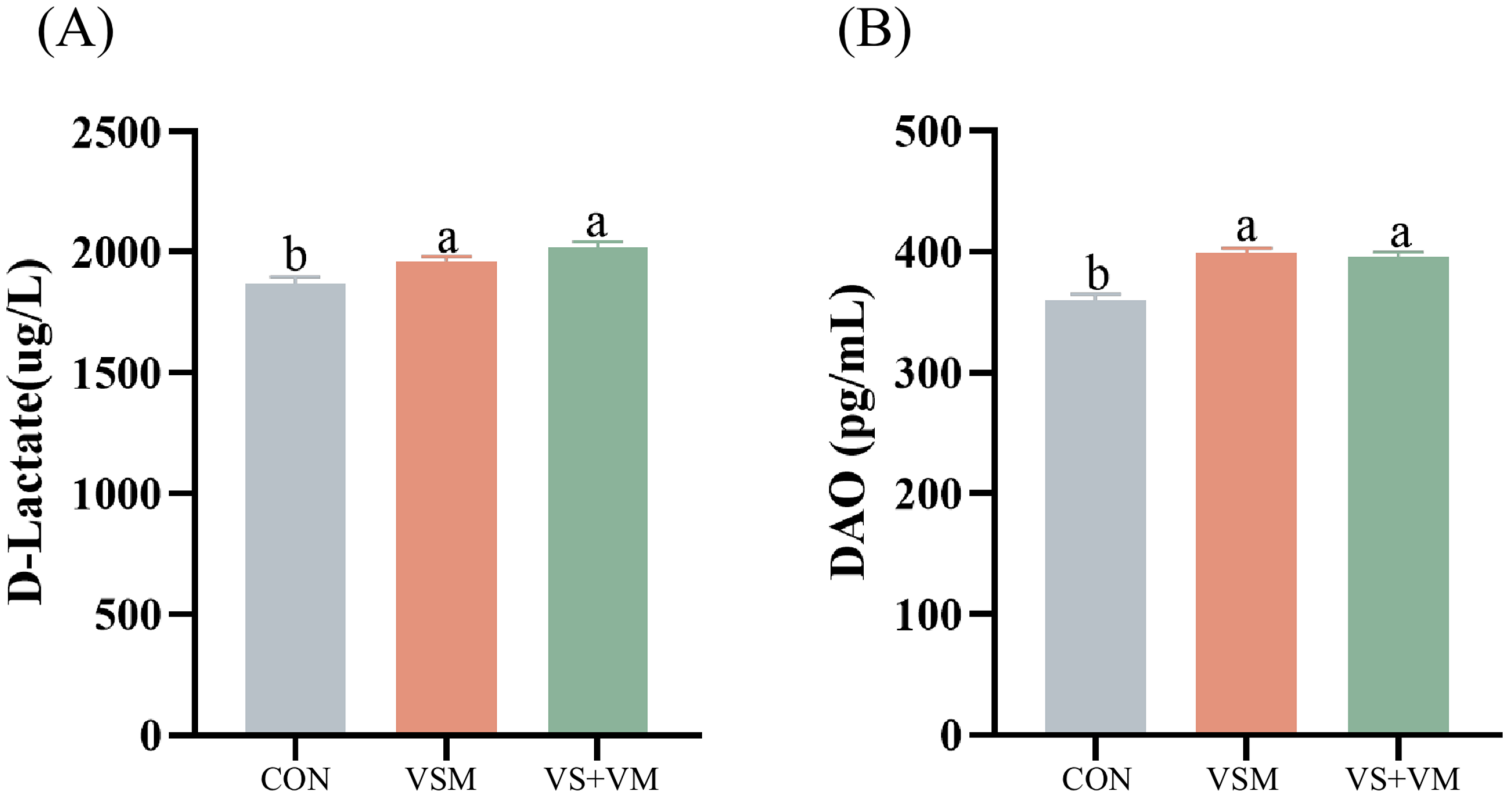
The effect of MCFA and SCFA on the concentrations of D-lactate and DAO in serum of weaning piglets. **(A)** D-lactic acid **(B)** DAO (diamine oxidase). CON, basal diet + ZnO/common organic acid; VSM, basal diet + higher MCFA and lower SCFA content; VS + VM, basal diet + higher SCFA and lower MCFA content. Data were shown as means ± SEM (*n* = 8). Different letters mean statistically significant difference among the groups (*p* < 0.05).

As shown in Figure 2, the intestinal permeability of weaned piglets is reflected by the serum levels of D-lactate and DAO, which are higher in the VSM group and the VS + VM group compared to the CON group (*p* < 0.05).

### Antioxidant stress

**Figure 3:**
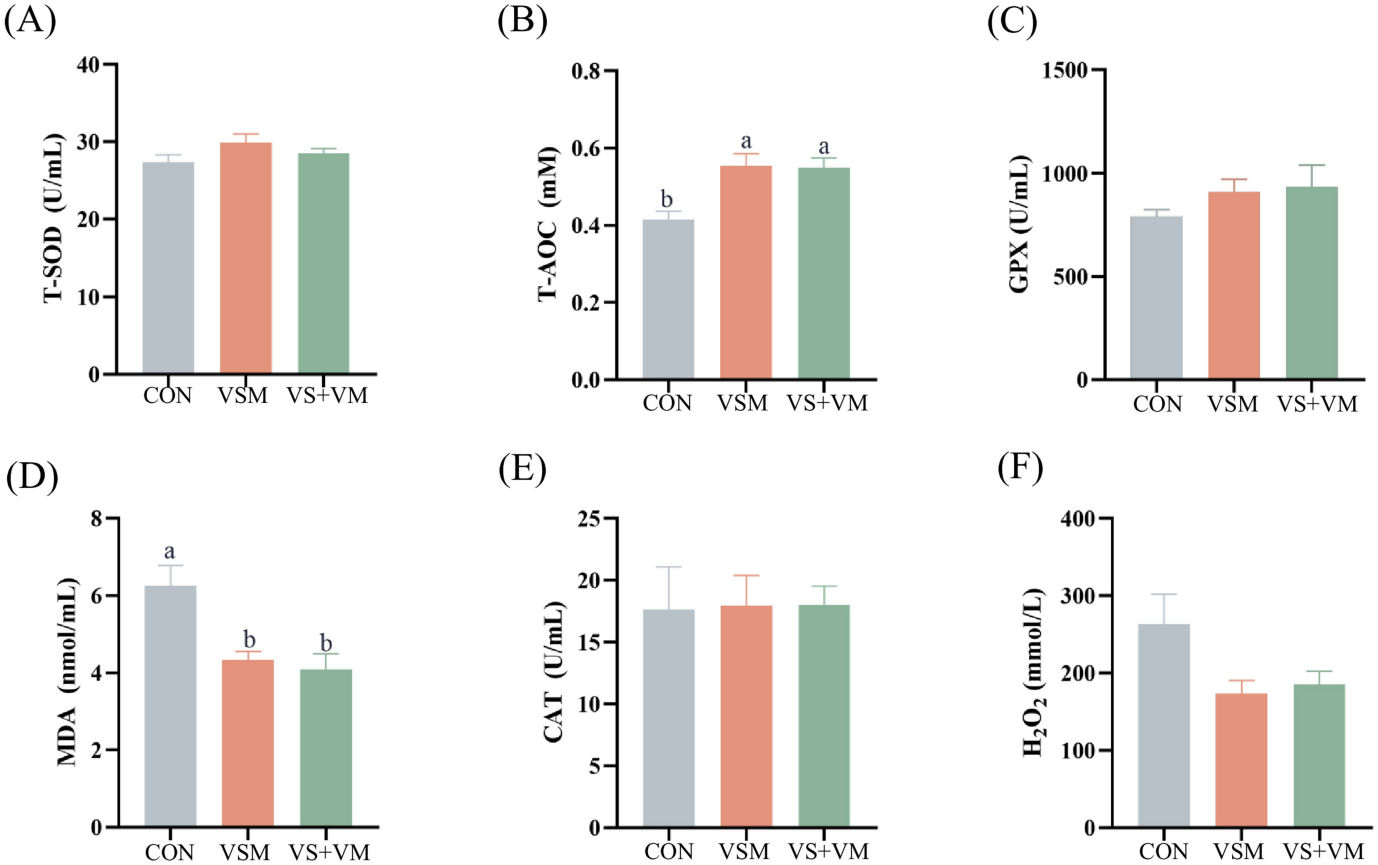
The effect of MCFA and SCFA on the serum antioxidant indicators. Serum levels of indicators including **(A)** T-SOD **(B)** T-AOC, **(C)** GPX, **(D)** MDA, **(E)** CAT, and **(F)** H_2_O_2_, were determined by using respective kits. T-SOD, total superoxide dismutase; T-AOC, total antioxidant capacity; GPX, glutathione peroxidase; CAT, catalase; MDA, malondialdehyde; H_2_O_2_, hydrogen peroxide CON, basal diet + ZnO/common organic acid; VSM, basal diet + higher MCFA and lower SCFA content; VS + VM, basal diet + higher SCFA and lower MCFA content. Data were shown as means ± SEM (*n* = 8). Different letters mean statistically significant difference among the groups (*p* < 0.05).

Antioxidant biochemical parameters in serum are presented in Figure 3. Compared with the CON group, both the VSM and VS + VM groups had significantly higher T-AOC levels in serum, and notably lower MDA concentrations (*p* < 0.05).

### Inflammatory cytokines

**Figure 4:**
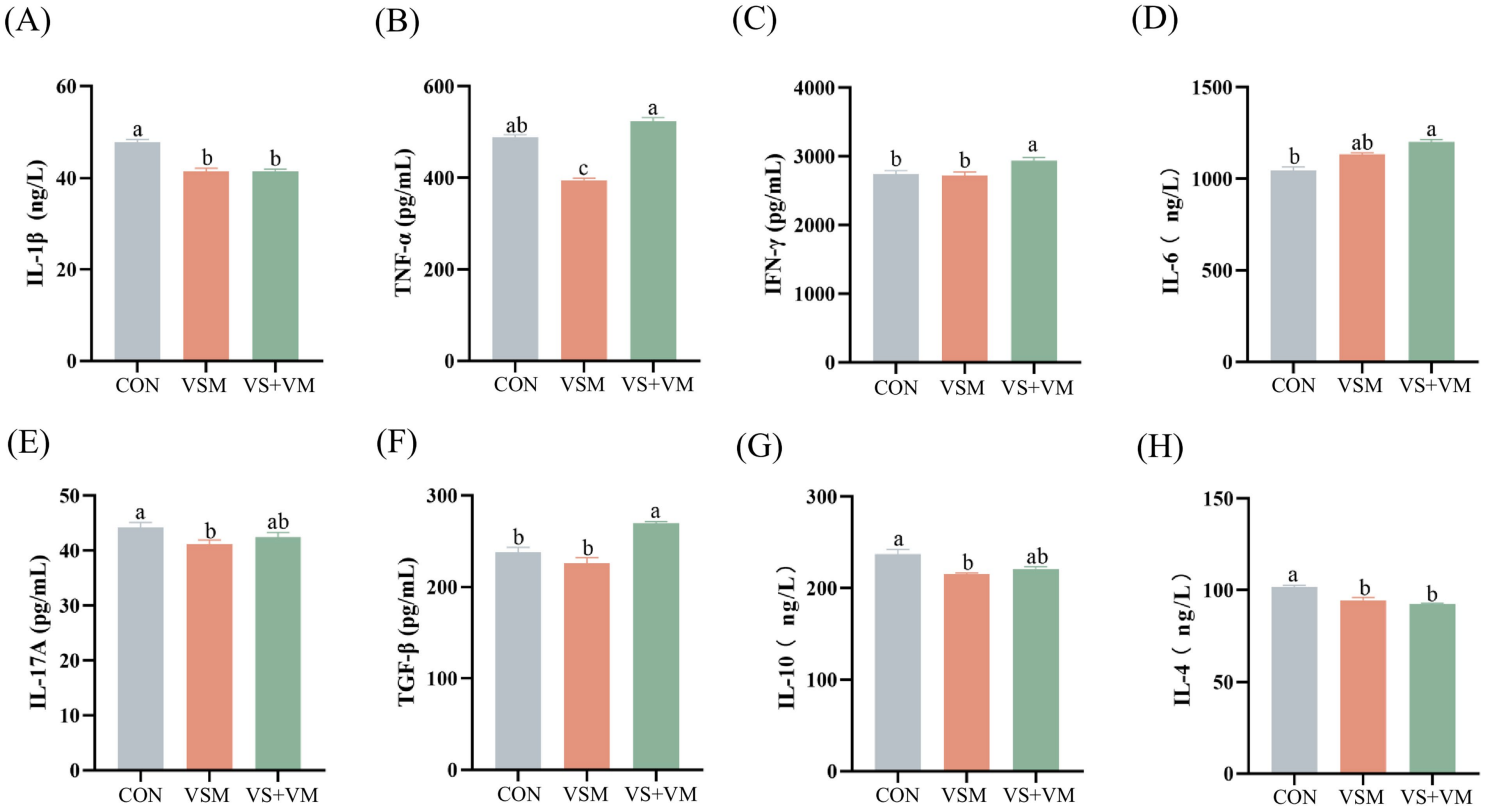
The effect of MCFA and SCFA on the production of inflammatory cytokines. Serum levels of **(A)** IL-1β, **(B)** TNF-α, **(C)** IFN-γ, **(D)** IL-6, **(E)** IL-17A **(F)** TGF-β **(G)** IL-10, and **(H)** IL-4 were measured by using ELISA kits. IL-1β, interleukin-1β; TNF-α, tumor necrosis factor-α; IFN-γ, interferon-γ; IL-6, interleukin-6; IL-17A, interleukin-17A; TGF-β, transforming growth factor-β; IL-10, interleukin-10; IL-4, interleukin-4. CON, basal diet + ZnO/common organic acid; VSM, basal diet + higher MCFA and lower SCFA content; VS + VM, basal diet + higher SCFA and lower MCFA content. Data were shown as means ± SEM (*n* = 8). Different letters mean statistically significant difference among the groups (*p* < 0.05).

The pro-inflammatory and anti-inflammatory cytokine profiles are presented in Figure 4. Compared with CON group, the VSM group notably reduced serum inflammatory cytokines, including IL-1β, TNF-α, IL-17A, IL-4, and IL-10 (*p* < 0.05). Thus, the VS + VM group significantly decreased IL-1β and IL-4, and increased IFN-γ, IL-6 and TGF-β in serum (*p* < 0.05).

### Gut microbiota community

**Figure 5:**
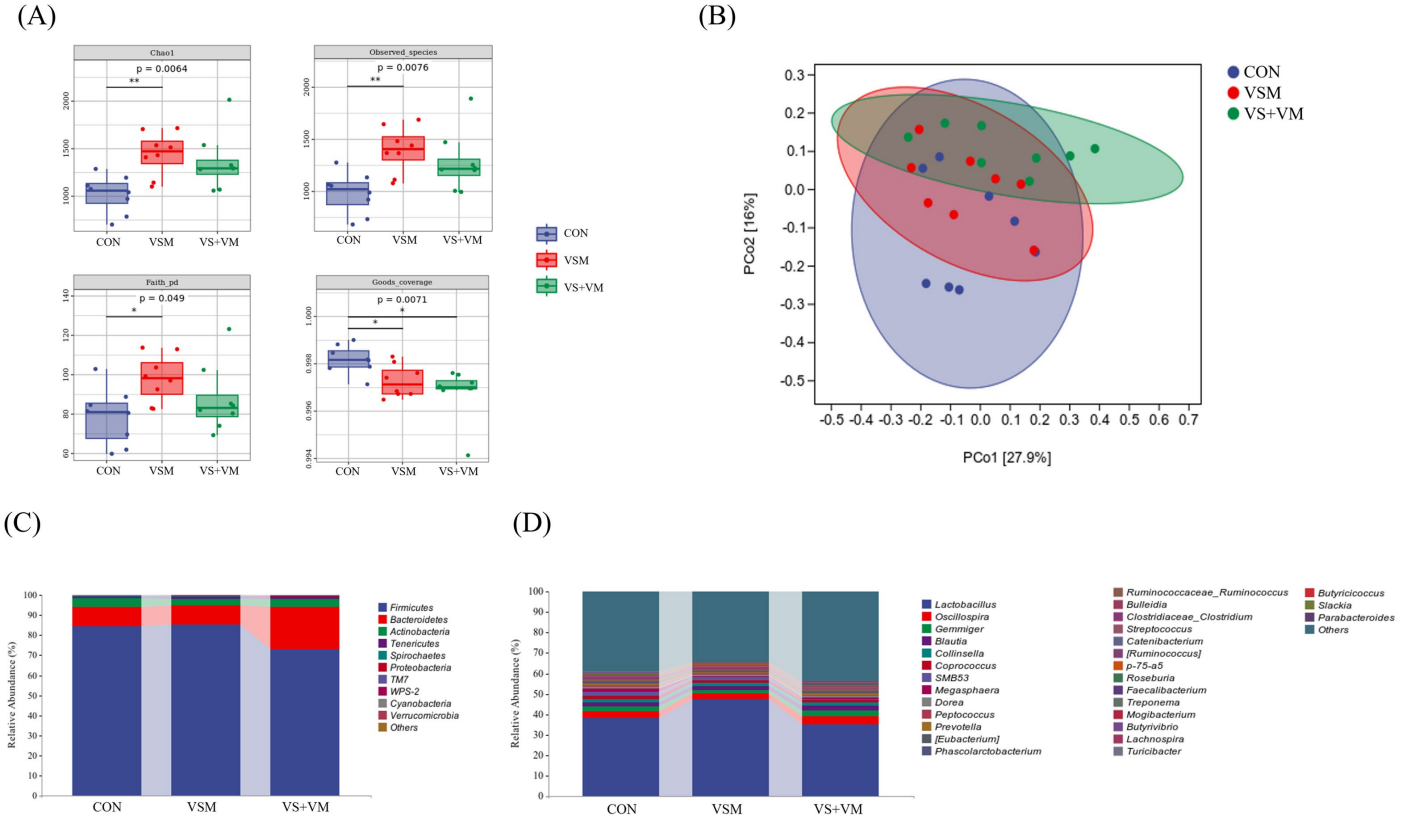
The effect of MCFA and SCFA of gut microbiota of piglets (*n* = 8) **(A)** Comparison of alpha diversity index (Chao1, Observed species, Faith PD, and Goods coverage). **(B)** Principal coordinates analysis (PCoA) based on Bray–Curtis **(C)** Relative abundance of bacterial phylum level. **(D)** Relative abundance of bacterial genus level. CON, basal diet + ZnO/common organic acid; VSM, basal diet + higher MCFA and lower SCFA content; VS + VM, basal diet + higher SCFA and lower MCFA content. Different letters mean statistically significant difference among the groups (*p* < 0.05) (^*^p < 0.05 and ^**^p < 0.01).

**Figure 6:**
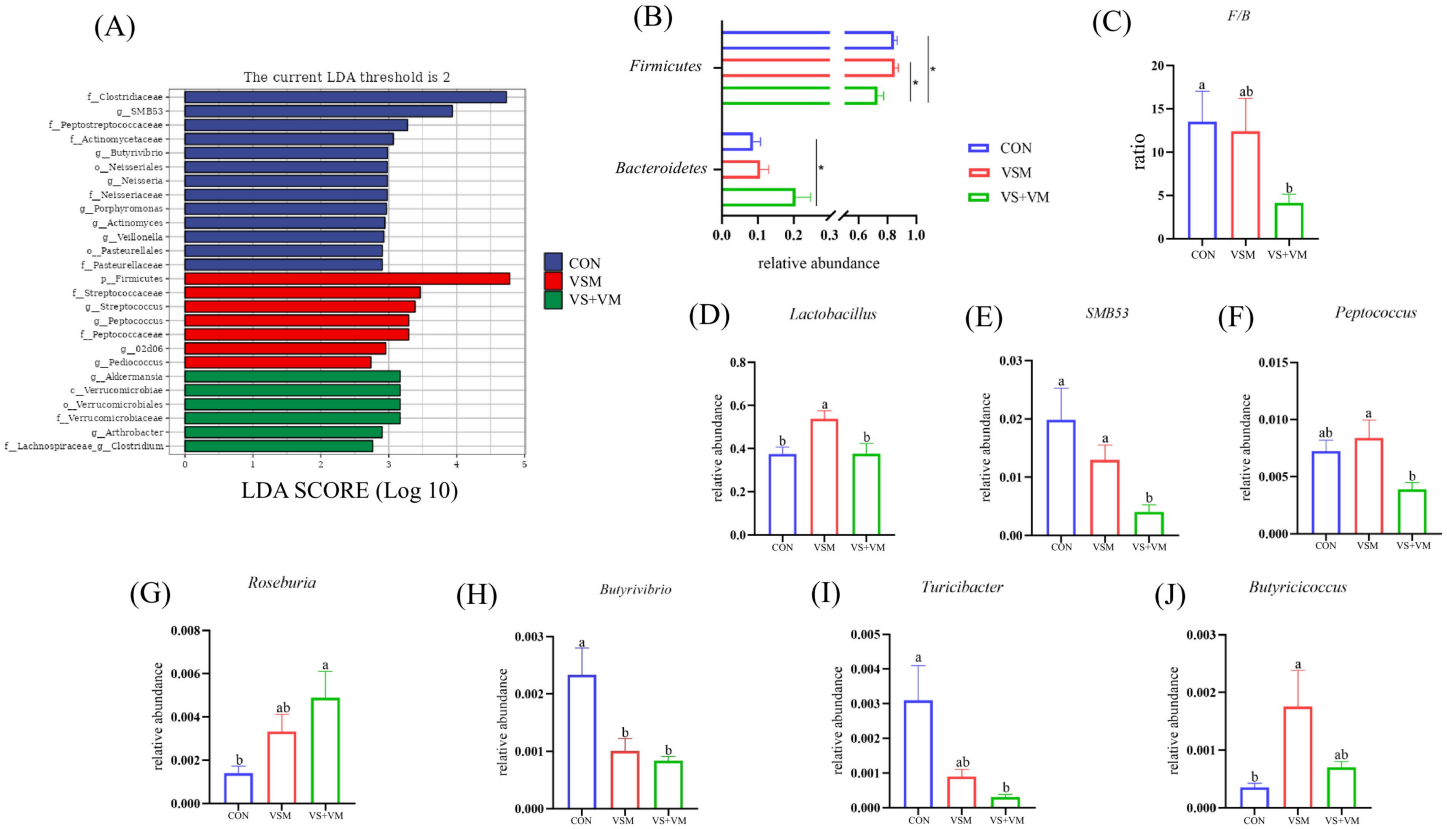
Effect of MCFA and SCFA of gut microbiota of piglets (*n* = 8). **(A)** LEfSe analysis effect size identified the most differentially abundant taxa in the cecal chyme microbiota community of each group, and only taxonomies of LDA score >2 is shown. **(B)** Relative abundance of *Firmicutes* and *Bacteroidetes* among the three groups. **(C)** The ratio of *Firmicutes* and *Bacteroidetes*. **(D–J)** Extended error bar plot showing the bacteria at the genus level that had significant. CON, basal diet + ZnO/common organic acid; VSM, basal diet + higher MCFA and lower SCFA content; VS + VM, basal diet + higher SCFA and lower MCFA content. Different letters mean statistically significant difference among the groups (*p* < 0.05) (^*^p < 0.05 and ^**^p < 0.01).

Gut microbes play a critical role in intestinal inflammation, we conducted 16S rDNA sequencing analysis of the intestinal bacteria. As shown in Figure 5, compared with the CON group, Chao1, Observed species, and Faith PD indices were significantly increased in the VSM group (*p* < 0.05); both VSM and VS + VM significantly decreased the goods coverage indices compared to the CON group (*p* < 0.05); At the phylum level, compared with the CON group, VS + VM group increased the relative abundance of *Bacteroidetes*, but decreased the relative abundance of *Firmicutes* and the ratio of *Firmicutes* to *Bacteroidetes* (*p* < 0.05) (Figures 6B,C). At the genus level, the abundances of genera *Lactobacillus* and *Butyricicoccus* were upregulated in the VSM group, and the abundance of genus *Butyrivibrio* were downregulated compared to the CON group (*p* < 0.05) (Figures 6D,H,J); further, the abundances of genera *Roseburia* was upregulated in the VS + VM group, and the abundances of genera *SMB53*, *Butyrivibrio* and *Turicibacter* were downregulated compared to the CON group (*p* < 0.05) (Figures 6E,G–I). The microbial composition of fecal samples was further analyzed by Linear discriminant analysis Effect Size (LEfSe) (Figure 6A). Thus, a total of 26 bacterial taxa were identified to be significantly different between the three groups.

### Spearman’s correlation analysis

**Figure 7:**
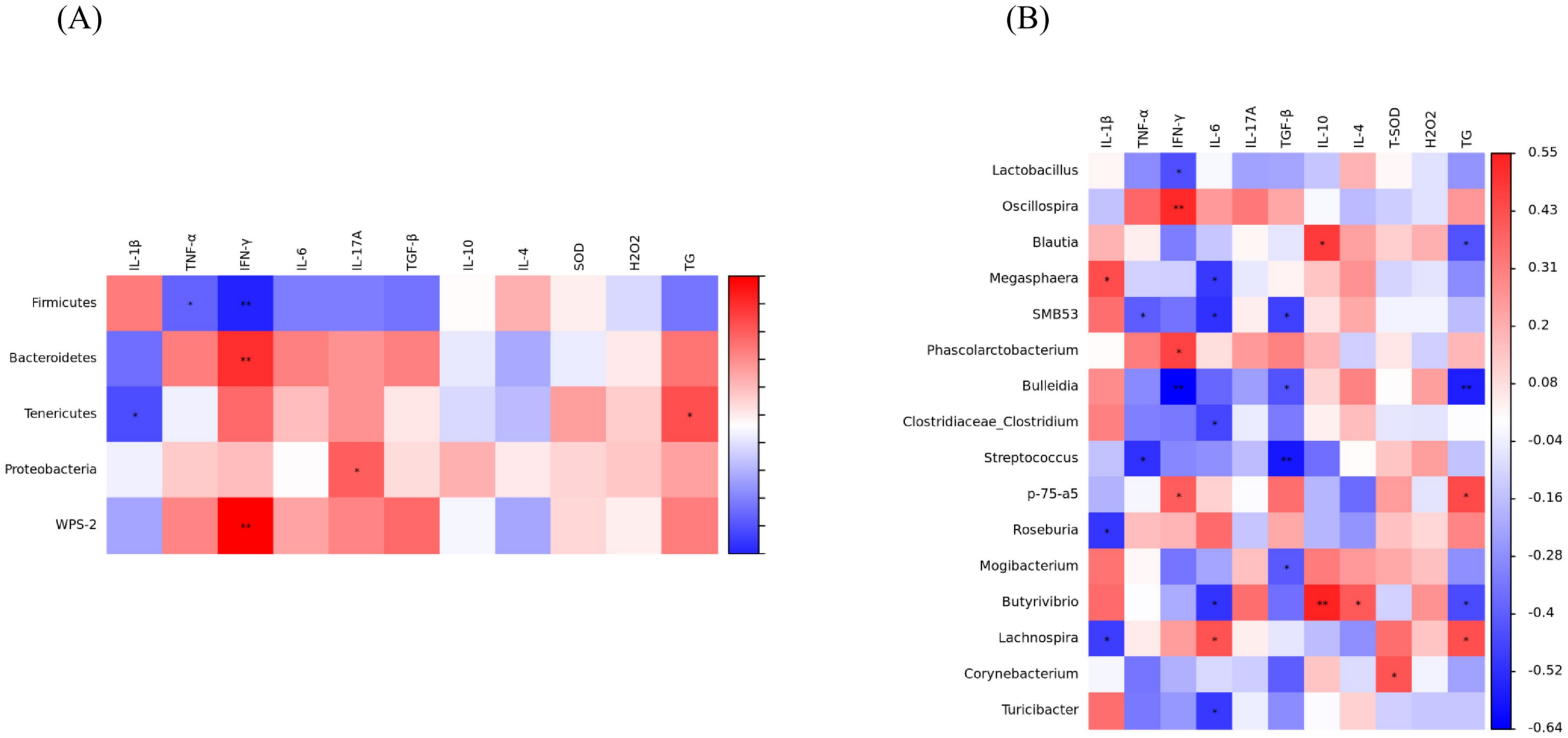
Spearman’s correlation between antioxidant indicators, inflammatory cytokines and gut microbiota. **(A)** Phylum. **(B)** Genus. IL-1β, interleukin-1β; TNF-α, tumor necrosis factor-α; IFN-γ, interferon-γ; IL-6, interleukin-6; IL-17A, interleukin-17A; TGF-β, transforming growth factor-β; IL-4, interleukin-4; IL-10, interleukin-10; T-SOD, total superoxide dismutase; H_2_O_2_, hydrogen peroxide; TC, total cholesterol. (Significant correlations were marked by ^*^p < 0.05 and ^**^p < 0.01).

The results of Spearman’s correlation analysis between gut microbiota and serum immune cytokines are shown in Figure 7. At the phylum level, *Firmicutes* showed negatively correlations with TNF-α and IFN-γ (*p* < 0.05). *Bacteroidota* exhibited a positive correlation with IFN-γ (*p* < 0.05). *Tenericutes* showed negatively correlations with IL-1β, whereas positively correlated with TG (*p* < 0.05). *Proteobacteria* was positively correlated with IL-17A and *WPS-2* was positively correlated with IFN-γ (*p* < 0.05) (Figure 7A). At the genus level, *Megasphaera* was positively correlated with IL-1β, *Roseburia* and *Lachnospira* showed a negative correlation with IL-1β (*p* < 0.05), *SMB53* and *Streptococcus* were negatively correlated with TNF-α (*p* < 0.05), *Oscillospira*, *Phascolarctobacterium* and *p-75-a5* showed positive correlations with IFN-γ (*p* < 0.05), whereas *Lactobacillus* and *Bulleidia* were negatively correlated with IFN-γ (*p* < 0.05), *Lachnospira* was positively correlated with IL-6 (*p* < 0.05), *Megasphaera*, *SMB53*, *Clostridiaceae_Clostridium*, *Butyrivibrio* and *Turicibacter* exhibited a negative correlation with IL-6 (*p* < 0.05); Additionally, *SMB53*, *Bulleidia*, *Streptococcus*, and *Mogibacterium* showed a negative correlation with TGF-β (*p* < 0.05), *Blautia* and *Butyrivibrio* were positively correlated with IL-10, but negatively correlated with TG (*p* < 0.05), *Butyrivibrio* was positively correlated to IL-4 and *Corynebacterium* was positively correlated with T-SOD (*p* < 0.05), *p-75-a5* and *Lachnospira* showed a positive correlation with TG, *Bulleidia* was negatively correlated with TG (*p* < 0.05) (Figure 7B).

## Discussion

MCFA and SCFA serve not only as energy sources for intestinal epithelial cells but enhance gut health through their broad-spectrum antibacterial activity (Lauridsen, 2020). By modulating the gastrointestinal microbiota, alleviating oxidative stress, and enhancing growth performance, MCFA and SCFA demonstrate considerable potential as alternatives to ZnO. Previous studies have found that the organic acids and MCFA exert a stronger growth-promoting effect on the weaned piglets than ZnO (Kuang et al., 2015). Additionally, combinations of SCFA and capric acid have been shown to improve production performance in weaned piglets and nursery pigs, likely by enhancing the digestibility of nutrients and supporting the structural integrity of the small intestinal mucosa (Hanczakowska et al., 2013). In this study, two dietary formulations with varied SCFA/MCFA ratio were analyzed, the proportion of MCFA was higher than that of SCFA in the VSM group, whereas the proportion of MCFA was lower than that of SCFA in the VS + VM group. Compared to the ZnO-based diet, both different ratios of MCFA and SCFA increased the ADG of weaned piglets in the first stage and reduced the F/G, aligning with previous findings and supporting the potential of fatty acids as ZnO replacements. However, in the second stage, the growth performance in the VS + VM group declined, suggesting that prolonged feeding of high formic acid levels may adversely affect feed intake, possibly due to changes in palatability. Throughout the entire experimental period, a higher MCFA proportion relative to SCFA appeared to improve the EE digestibility, with a more pronounced positive effect on growth performance.

Serum biochemical parameters reflect the metabolic level and health status of the animal body. In the present study, MCFA and SCFA did not alter TP and BUN content, which was similar to the CP digestibility results, indicating that MCFA and SCFA have no impact on the utilization and metabolism of proteins. Furthermore, serum levels of TG were elevated in the VS + VM group, which was similar to an earlier study (Cera et al., 1989), this phenomenon may be attributed to fatty acids serving as energy substrates to enhance TG synthesis, However, several studies have reported that MCFA can reduce serum TG levels (van de Heijning et al., 2017), and the exact mechanism needs to be further explored.

Oxidative stress is a primary cause of barrier damage and gastrointestinal diseases in piglets, often resulting from an excess of ROS that surpasses exceeding the antioxidant capacity (Lee and Kang, 2017; Qiao et al., 2023). Organic acids help maintain redox homeostasis, and previous studies have indicated that organic acid-based products, such as formic and acetic acid, can alleviate oxidative stress in intestinal epithelial cells (Xu et al., 2022). It is well-known that T-AOC reflects the overall ability of a biological system to counteract oxidative stress by neutralizing free radicals. In contrast, MDA is a byproduct of lipid peroxidation and serves as an indicator of oxidative damage within cells. In the current study, both of MCFA groups showed increased T-AOC level and decreased the MDA levels compared to the ZnO control, consistent with previous study (Long et al., 2018). It was demonstrated that dietary MCFA and SCFA improve antioxidant capacity, suggesting they can alleviate weaning-induced oxidative stress in piglets.

The intestinal barrier selectively facilitates the absorption of nutrients while serving as an important defense mechanism to prevent the translocation of bacteria and toxins from the intestinal lumen to other tissues. Weaning stress compromises the physical barrier, characterized by damage to tight junctions in the intestinal epithelium and increased intestinal permeability (Tang et al., 2022), potentially promoting bacterial translocation and exposing other tissues to pathogenic threats. When intestinal permeability increases, DAO secreted by intestinal mucosal cells and D-lactic acid, a product of intestinal bacteria, can enter the bloodstream through the intestinal mucosa. Consequently, these markers are widely used to evaluate intestinal permeability (Song et al., 2013). In the present study, dietary supplementation with MCFA and SCFA resulted in higher serum concentration of DAO and D-lactic acid, suggesting that ZnO may be more effective in reducing intestinal permeability. Previous research showed that ZnO could reduce the serum levels of DAO and D-lactic acid in piglets (Peng et al., 2019). Further investigation is warranted to elucidate the potential of MCFA and SCFA in improving intestinal permeability.

The gut microbiota represents another essential barrier, whose dynamic equilibrium formed by their interdependence and mutual restriction plays a crucial role in maintaining the host’s intestinal immune function. In the early stages of weaning, piglets are unable to fully utilize the nutrients in their diet, which provides a source of nutrition for the growth of pathogenic bacteria and may lead to the disruption of the microbial barrier (Su et al., 2022), underscoring the need for nutritional interventions to maintain gut microbiota balance (Lim et al., 2023). MCFA and SCFA exhibit certain antibacterial effects. Undissociated fatty acids penetrate bacterial membranes and dissociate within the alkaline cytoplasm of pathogens such as *Escherichia coli*. Releasing protons that acidify the intracellular environment. This acidification disrupts essential metabolic pathways and ultimately induces bacterial cell death (Gomez-Osorio et al., 2021). *Lactobacillus* and other beneficial bacteria have acid-resistant characteristics. Additionally, an appropriate concentration of acid can serve as a nutritional source for *Lactobacillus* (Hsiao and Siebert, 1999). Previous studies have found that supplementation of sows’ diets with a mixture of organic acids and MCFA increases fecal counts of *Lactobacillus* and decreases *Escherichia coli* counts at farrowing and weaning (Lan and Kim, 2018). Our study found that the relative abundance of *Lactobacillus* was significantly higher in the VSM group, in addition, the indices of Chao1 and Observed species were improved in the VSM group. This indicates that a combination with a higher proportion of MCFA than SCFA shows better performance in selectively inhibiting harmful bacteria and promoting the growth of beneficial bacteria.

Dysbiosis in the gut microbiome, characterized by reduced microbial diversity and increased pathogenic bacteria, is closely related to the host inflammatory response (Bäumler and Sperandio, 2016). Dietary supplementation with specific fatty acids, such as MCFA and SCFA, may represent a promising strategy for mitigating intestinal inflammation in piglets (Liu, 2015). Previous studies have found that dietary supplementation with sodium caprylate enhanced immune function by regulating the gut microbiota and increasing the intestinal SCFA concentrations in mice (Zhao et al., 2021). In this study, a higher proportion of MCFA relative to SCFA not only improved microbiota richness and abundance of *Lactobacillus* but also reduced pro-inflammatory cytokine levels, including TNF-α, IL-1β, and IL-17A. These findings suggest that an optimal MCFA-to-SCFA ratio may alleviate inflammatory responses by modulating the gut microbiota. Furthermore, in this study, supplementation with MCFA and SCFA increased the relative abundance of *Roseburia* while decreasing the relative abundance of *Turicibacter*. *Roseburia* has been shown to exert protective effects against gut-dysbiosis-induced mastitis, mucosal inflammation, and systemic inflammation (Zhao et al., 2022). A correlation analusis revealed a negative association between *Roseburia* and IL-1β, a pro-inflammatory factor implicated in intestinal inflammation (Kaminsky et al., 2021), supporting the anti-inflammatory potential of *Roseburia*. Conversely, the pathogenic bacterium *Turicibacter* has been reported to increase in colitis mouse model (Wan et al., 2021). Interestingly, while *Butyrivibrio* bacteria, known for fermenting dietary fibers to produce SCFA like butyrate (Kopečný et al., 2003), decreased in MCFA and SCFA supplementation, this aligns with previous studies attributing the reduction to negative feedback regulation induced by exogenous SCFA supplementation (Zhou et al., 2017). Collectively, these findings indicate that supplementation of MCFA and SCFA positively regulate the intestinal microbiota composition, contributing to the improvement of intestinal health.

## Conclusion

In this study, diets with a higher MCFA to SCFA ratio (VSM group) exerted more favorable effects on piglet growth performance and exhibited promise as a potential alternative to zinc oxide. These benefits were associated with improvements in gut microbiota composition, enhanced antioxidant capacity, and reduced inflammatory responses. However, the results for the VS + VM group, where SCFA levels exceeded MCFA, were less conclusive in terms of growth promotion. This may be attributed to the high formic acid content in the SCFA formulation, which could have affected feed palatability and consequently limited nutrient intake. While VS + VM supplementation exhibited some positive effects on gut health and inflammatory modulation, its overall impact on growth performance warrants further investigation.

## Data Availability

The raw data for all 16S rRNA amplicons sequenced in this study have been deposited in NCBI’s Sequence Read Archive (SRA) under accession number PRJNA1243349.

## Glossary

ADFI: Average daily feed intake
ADG: Average daily gain
ALB: Albumin
ALP: Alkaline phosphatase
AIA: Acid-insoluble ash
Ash: Crude ash
ATTD: Apparent total tract digestibility
BUN: Blood urea nitrogen
BW: Body weight
Ca: Calcium
CAT: Catalase
CP: Crude protein
DAO: Diamine oxidase
EE: Crude fat
ELISA: Enzyme-linked immunosorbent assay
F/G: Feed to gain ratio
GLB: Globulin
GLU: Glucose
GPX: Glutathione peroxidase
H_2_O_2_: Hydrogen peroxide
IFN-γ: Interferon-γ
IL-1β: Interleukin-1β
IL-4: Interleukin-4
IL-6: Interleukin-6
IL-10: Interleukin-10
IL-17A: Interleukin-17A
MCFA: Medium-chain fatty acid
MDA: Malondialdehyde
P: Phosphorus
ROS: Reactive oxygen species
SCFA: Short-chain fatty acid
T-AOC: Total antioxidant capacity
TC: Total cholesterol
TG: Total glyceride
TGF-β: Transforming growth factor-β
TP: Total protein
T-SOD: Total superoxide dismutase
TNF-α: Tumor necrosis factor-α
ZnO: Zinc oxide
